# Industry 1.61803: the transition to an industry with reduced material demand fit for a low carbon future

**DOI:** 10.1098/rsta.2016.0361

**Published:** 2017-05-01

**Authors:** Julian M. Allwood, Timothy G. Gutowski, André C. Serrenho, Alexandra C. H. Skelton, Ernst Worrell

**Affiliations:** 1 Department of Engineering, University of Cambridge, Trumpington Street, Cambridge CB2 1PZ, UK; 2 Department of Mechanical Engineering, Massachusetts Institute of Technology, 77 Massachusetts Avenue, Cambridge, MA 02139, USA; 3 Copernicus Institute of Sustainable Development, Heidelberglaan 2, 3548 Utrecht, The Netherlands

**Keywords:** climate change mitigation, material demand reduction, transition, policy

## Abstract

Arising from a discussion meeting in September 2016, this editorial introduces a special issue on the transition to a future industrial system with greatly reduced demand for material production and attempts to synthesize the main findings. The motivation for such a transition is to reduce industrial greenhouse gas emissions, but unlike previous industrial transformations, there are no major stakeholders who will pursue the change for their own immediate benefit. The special issue, therefore, explores the means by which such a transition could be brought about. The editorial presents an overview of the opportunities identified in the papers of the volume, presents examples of actions that can be taken today to begin the process of change and concludes with an agenda for research that might support a rapid acceleration in the rate of change.

This article is part of the themed issue ‘Material demand reduction’.

## 1. Introduction: past and future systems of industrial organization

Manufacturing began as craft but even by the third millennium BC, craftsmen had developed tools and systems to achieve economies of scale. Gold beakers found in the Royal Tombs of Ur in Sumeria from 2600 BC demonstrate repeating patterns made by specially shaped punches [1] and the coins from Lydia in the seventh century BC are punched with dies whose intricate patterns are a guarantee of authenticity. Every generation since has sought to reduce the costs of goods by augmenting the productivity of labour and seeking scale economies through using the latest technology to substitute for individual craft skills. The Persian armourers of the fifteenth century organized their production of mail shirts with up to 30 stages from wire drawing, ring formation, joining and finishing [2] and the industrial revolution began in England in the eighteenth century when the wide availability of cheap fossil-fuel energy triggered a broad substitution of energy (and capital) for labour. The ubiquitous mechanization of material and component production in the twentieth century focused the minds of manufacturing managers on the efficiency of labour in assembling components into products. Frederick Taylor's ‘Principles of scientific management’ sought a new level of productive efficiency by eliminating human choice from shop floor operations [3] and this was extended with Henry Ford's invention of the continuous chain assembly line [4]. The attempt to reduce work to an unvarying recipe proved to be a poor means to cope with variability in production. Japanese managers, therefore, worked through the ‘Just-in-Time’ revolution, which aimed to make unexpected variations more immediately apparent. This culminated in the now widely copied ‘Toyota Production System’ which aims to train workers to identify and diagnose problems in order to eliminate their root cause [5].

In the richer economies, prior to around 1950–1960, these developments in manufacturing were motivated by the commercial imperative to turn luxury products into commodities. High cost products that could be purchased only by the wealthy few could be sold to mass markets as their costs fell. Henry Ford's ambition to pay his workers sufficiently and to make a car so cheaply that it could be purchased by the people who made it was translated across all products. By around 1960, virtually all consumers in the developed economies could purchase a version of any product, so manufacturers turned their attention to creating variety and innovation: variety allowed market segmentation, so wealthier consumers would pay more; innovation created dissatisfaction with existing goods, so rather than expanding the market for existing products, manufacturers targeted replacement purchasing.

The Toyota Production System has proved effective in organizing labour to cope with controlled variations around template products (mass customization) but the costs of production now are influenced more by those associated with design, marketing and introducing new products than in mass replication. Accordingly, twenty-first-century manufacturing managers aim to apply the techniques developed for efficient assembly during the twentieth century to the processes of new product innovation where speed of introduction is more critical than the optimization of long-term steady-state production. Today's focus is on the application of rapidly developing information technology to the connected processes of marketing, innovation and production and in particular to exploiting the information seams created by the Internet. The widely touted ‘Internet of Things’ which anticipates a great expansion in the range of products that can be addressed remotely combined with excitement about the possibility of automated statistical pattern recognition applied to ‘Big Data’ leads to contemporary interest in ‘Industry 4.0’ [6]. This emerging approach to industrial organization anticipates a rapid expansion in the automation of innovation connected to manufacturing.

However, in parallel with this focus on automated innovation as the next step in the ‘normal business’ of stimulating demand to maximize revenues, global demand for ever-more energy supplied by fossil fuels is driving global warming. The rapid effects of climate change during the twenty-first century are certain to enforce a significant change to ‘normal’ patterns of business, with adaptation and survival acting as parallel motivations to existing metrics of profit. Climate change is much discussed, domestically, commercially and politically, but in reality little significant action has occurred. Globally, greenhouse gas emissions continue to rise and at an ever-faster rate.

The question of how we respond to climate change turns fundamentally on the question of whether an alternative to fossil fuels can be found to provide power at levels equivalent to today's global demand. The next section demonstrates that this is unlikely, so mitigation depends on the demand reduction options described in §1b. These have had little priority in policy discussions to date which leads to the motivation for this special issue set out in §1c.

### (a) Low carbon energy supply options will not be sufficient to mitigate climate change

Where action on climate change is happening it is strongly focused on the search for an alternative energy supply. This search has revealed three key options, yet it is unlikely that they will deliver today's level of global energy consumption or be deployed sufficiently rapidly to avoid the dangerous levels of warming that policy aims to avoid:

— public sentiment has turned away from nuclear power, and global nuclear output has declined since 2007 [7].
— carbon capture and storage (CCS) has been deployed for enhanced oil recovery and at one pilot scale power plant in Canada, yet even the optimistic Global CCS institute funded by the oil and gas industry can only claim that the total maximum capacity of CCS installed by 2015 was 28 Mt, compared with global anthropogenic emissions of 51 000 Mt [8]. Within the UK, planned support for the commercialization of CCS (including up to £1 billion in capital funding and continued operational support through Contracts for Difference) was withdrawn on budgetary grounds in 2015 [9]. This was despite strong recommendations in the IPCC's most recent assessment report that limiting warming below 2°C would require net negative emissions in the second half of the century [10].
— the cost of ‘new’ renewable generation (wind turbines, biofuels and solar cells) is falling, but these are low-density supplies which must be deployed over large land areas. For countries, such as those in Western Europe, with a relatively high population density, it is unlikely that sufficient land area can be committed to allow energy generation at today's level of demand [11]. The political complexity of electricity transmission from desert areas to populous countries is as yet untested.

**Figure 1.**
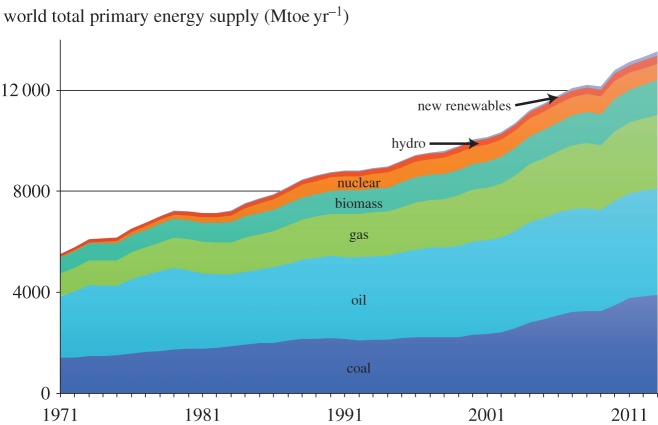
Total global energy supply in units of ‘Mega tonnes of oil equivalent’ [12].

As a result, the latest data from the International Energy Agency in figure 1 show that nuclear power delivers only approximately 5% and new renewables approximately 1.5% of our total current needs, while global consumption of the fossil fuels is still expanding. Many institutions have published scenarios projecting a radically different future energy supply mix. However, these scenarios are predicated on assumptions about political and commercial will and are generally extremely optimistic about relatively young technologies. As a counterbalance to this optimism, if the trends in figure 1 continue roughly linearly then by 2050, total energy demand will have expanded by around 50% and only approximately 7% of it will be supplied by low carbon sources. This pragmatic assessment is not to deny that alternative energy supplies should be developed as vigorously as possible as a key component of climate mitigation. However, over-confidence about the scale of alternative energy supplies is a major inhibitor to other components of the required strategy. Only when it is widely understood that alternative energy supplies will deliver much less energy than is used today will it be a priority to develop other actions.

### (b) Mitigation requires reductions in demand for energy and materials

A consequence of facing up to the inevitable limits to a future transformed energy supply is that a low carbon future must be a low-energy future [13]. Energy efficiency can make some contribution to this, but the manufacturers who produce motors and burners already compete on the efficiency of their offerings. Motor sport, for example, is predicated on maximizing engine efficiency and there are few significant gains remaining. Within the energy intensive industries, those that produce the bulk materials (in particular, steel, cement, plastic, paper and aluminium) are already highly motivated to pursue efficiency in their use of energy, as energy is such a high fraction of their input costs. For steel makers, the cost of coking coal and other energy is around a third of the costs of making crude steel. At most there are potential savings of around 27% in the energy intensity of industry [14], but these would come at high cost and be slow to implement. Efficiency has, therefore, limited potential to mitigate climate change at the level of current targets if it is to be invisible to final consumers. However, if our objective was defined differently—for example, to deliver today's services in a different form or to find cost-effective means to achieve a collectively agreed set of societal goals—then there is tremendous potential for energy saving. The phrase ‘demand reduction’ will be used to describe these approaches which require public engagement, to draw a contrast with the pursuit of invisible ‘efficiency.’

The world record for automotive fuel consumption is held by the PACII car, which achieved in excess of 15 000 miles per gallon (0.0186 l per 100 km) through ultra-light weight design, a minimal frontal area and a low top speed [15]. This design is extreme, but if legislated or preferred by consumers, all car manufacturers could in the near future be selling 200 miles per gallon (1.4 l per 100 km) cars. Such cars would permit the same journeys as made today, but the users would notice a difference: the cars would be smaller, travel more slowly and have a reduced acceleration. Within this volume, Serrenho *et al*. [16] contrast the likely mitigation effects of either electrifying cars or reducing their weight. Their results clearly prioritize weight reduction, to reduce the total energy input required for personal mobility regardless of powertrain. This creates an increased opportunity for future electrification with a reduced total requirement for low carbon electricity generation. Similarly, if we chose to do so, we could live comfortably in buildings using 75% less energy for heating and cooling air and water [17], and we could reduce the emissions of our diet by eating much less red meat [18].

A low-energy industrial sector would be one that produces less of the bulk materials. Recycling materials, where possible, generally saves energy compared to making new materials, although for most materials recycling used products leads to a reduction in quality. Recent policy interest around a ‘Circular Economy’ encourages interest in the destiny of materials at the end of life of products but should be seen as a secondary priority compared with the wider energy saving option of designing products which last longer and use less new material [19]. In previous papers, the authors of this editorial have established the need for material demand reduction [20,21] and examined the technical options to live well with reduced material production [22–25]. There are myriad options for designing and making buildings, vehicles, equipment and other goods with half as much material and keeping them for twice as long. This would reduce material consumption and hence industrial emissions to around a quarter.

### (c) Motivation for this special issue

Although there is apparently a large technical potential for supply-side strategies to mitigate climate change, rates of deployment of nuclear and CCS options suggest this is unlikely to be realized and the scale of land area commitment required to implement renewables is daunting. On the demand side, the technical potential for mitigation is perhaps even larger and it would also certainly be possible to continue to live well but differently with 75% less energy *per capita* than in the developed economies today. However, the challenge of deploying supply and demand side options is quite different. To date governments and agencies such as the IPCC that advise them have given little priority to the demand side options for fear of political unpopularity. A key concern behind this special issue is that while policy discussions continue to focus almost exclusively on supply-side options, action on demand side measures is being delayed. A plausible trajectory towards a zero carbon future might be a 75% cut in developed economy energy demand *per capita* matched with growth of developing country energy use to this level, all to be supplied by expansion of the three non-emitting energy options described above. But policy to date has focused narrowly on only the supply part of this trajectory. Unlike the previous developments in industrial organization reviewed above, there are no strong stakeholder groups who want to promote reductions in material demand. Industry 4.0 is being pursued with vigour by incumbent industries (wanting to reduce costs and increase the speed of innovation) and supportive governments (wanting to create knowledge intensive barriers to international competition). But an industrial system with reduced material demand is not currently in any group's direct interest, although it is probably essential to human survival.

Accordingly, this special issue has been assembled to explore a different transition, to a postulated ‘Industry 1.61803’. This intentionally provocative title draws on the use of the famous ‘golden ratio’ as an indicator of balance, between proportions and between humans and their physical surroundings. We can imagine ‘Industry 1.61803’ as an industrial system in harmony with the requirements of the climate and one which balances human needs for employment and purpose with secure, comfortable, satisfying lifestyles. The special issue arises from a 3-day discussion meeting held in Cambridge in September 2016. The participants at the meeting were selected to represent a diverse spread of disciplinary interests (table 1) and in anticipation were supplied with some of the material summarized above and a trigger paper [26]. Prior to the meeting, each participant wrote a draft response to this material from their own disciplinary experience and at the meeting each paper in turn was discussed to attempt to draw out connections, contradictions and shared insights. Revised papers were then submitted and sent for independent peer review. This editorial attempts to present some overview of the new insights that emerged from these papers and the rich discussion among participants during the meeting. In doing so, it attempts to reach out beyond the meeting, to anticipate priority research questions and to explore options for actions that might begin the urgent process of transition to a future Industry 1.61803.

**Table 1.** Overview of attendee disciplines and topics discussed at the September 2016 workshop leading to this special issue.

disciplinary background	title of paper
social anthropology	Climate strategies: thinking through Arctic examples
engineering	Why we use more materials
theology and philosophy	Not to escape the world but to join it: responding to climate change with imagination not fantasy
design	Exploring demand reduction through design, durability and ‘usership’ of fashion clothes
innovation studies	Political economies and environmental futures for the sharing economy
engineering	The impact of reducing car weight on global emissions: the future fleet in Great Britain
social psychology	Who is reducing their material consumption and why?
economics	Are prices enough? The economics of material demand reduction
public health	Towards environmentally sustainable human behaviour: targeting non-conscious and conscious processes for effective and acceptable policies
business school	Frugal innovation: doing more with less for more
sociology	Why on earth did I buy that? A study of regretted appliance purchases
economics and engineering	The carbon price: a toothless tool for material efficiency?
media and environment	Demanding stories: television coverage of sustainability, climate change and material demand
social policy	Recomposing consumption: defining necessities for sustainable and equitable well-being
social psychology	The austere life
energy and resources	Energy demand for materials in an international context
political science	Living both well and sustainably
ecological economics	Radical dematerialization and de-growth

## 2. Insights into unlocking a transition to Industry 1.61803

No one currently lobbies to reduce material demand but the transition reduced material demand is essential if we are to mitigate the effects of climate change sufficiently. A convenient means to structure our investigation of this challenge is to consider the problem as an interaction among three major stakeholder groups:

— individuals, as consumers, employees and voters, who seek security, status and satisfaction in work and leisure;
— businesses, as employers and the recipients of investment, whose primary objective is to maximize their return to the financial investment that funded their establishment;
— governments, as the arbiters of taxation and regulation, whose ministers set policy aiming to balance competing interests while maintaining sufficient popularity to be re-elected.

**Figure 2.**
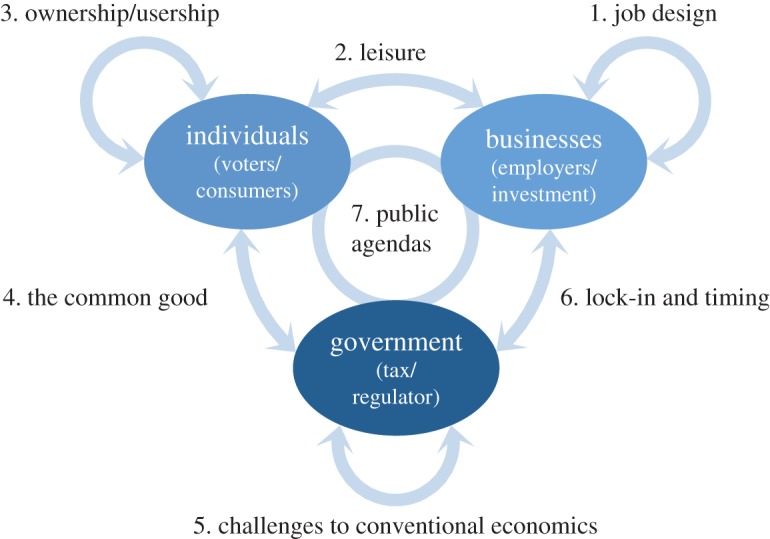
The three main stakeholder groups that influence material demand, and the seven interactions between them that define the structure of the main section of this paper. (Online version in colour.)

Figure 2 shows a simple schematic of these three groups, with seven interactions within and between them, which define the structure of the remainder of this section. Drawing on the papers in this issue, the section aims to develop insights into these stakeholder interactions which may be of value in identifying features of the transition to Industry 1.61803.

### (a) Businesses: job design

The economies of scale which introduced this article are unavoidable. On the supply side they originate from spreading capital costs and set-up times over larger output, standardization and learning-by-doing. For almost all products, the energy required for production also shows an economy of scale. Blast furnaces become more efficient as they become larger, as do electric motors and diesel engines. Even for products which are inherently unique such as commercial buildings, dedicated equipment or medical implants, producers find economies of scale by standardizing common features to reduce variability to as few features as possible. On the demand side, economies of scale arise from marketing and branding, trends and herd behaviour. These economies are restrained only by the limits to market tolerance for uniformity.

The economies of scale found in twentieth-century manufacturing developments have been beneficial in making products available to a much greater segment of society and in minimizing energy used per product. Yet these economies also create a loss which is clearly indicated in Frederick Taylor's brutal descriptions of manufacturing workers. ‘One of the very first requirements of a man who is fit to handle pig iron as a regular occupation is that he shall be so stupid and phlegmatic that he more nearly resembles in his mental make-up the ox than any other type’ [3]. In reaction to what he identified as the inefficiency of an ‘old-style management’ in which teams of workers should be given incentives to show initiative and improve their work, Taylor believed that all decisions about the planning and design of work tasks should be made by managers who always know more about how best to do things. In effect his system led to the creation of two parallel manufacturing systems: a physical system which delivered the product and a planning system in which the physical system was designed, monitored and improved. The two systems were staffed separately, with the second group exerting complete control over the first.

This division is obviously demeaning for the physical workers and management textbooks, therefore, describe how the activities of professional ‘human resource managers’ in the second half of the twentieth century have redesigned these ‘ox-like’ jobs to be more fulfilling. But, by contrast, Harry Braverman [27] in his highly quoted 1973 book ‘Labour and monopoly capital: the degradation of work in the twentieth century’ argues not just that Taylor's principles have pervaded the service industries and many other fields of work, but also that they are an inevitable outcome of capitalism. Building on Charles Babbage's 1832 ‘On the economy of machinery and manufactures’ [28] and Karl Marx's ‘Capital,’ Braverman argues that in a capitalist economy, ‘the purpose of employment is the expansion of a unit of capital belonging to an employer.’ The most efficient means of achieving this expansion is to follow Taylor's principles and remove all choices about work design from the worker. Therefore, according to Braverman, ‘struggles over job control are the central feature of work under capitalism.’

Marx established a contrast between a bee building a honeycomb and an architect designing and building a house. The bee builds because it knows no other action but the human architect first conceptualizes the design in his or her mind and subsequently executes it. This power to conceptualize as well as to execute is, according to Marx, the unique feature of humans. Not only do humans establish control over nature but the capacity for conceptualization is the most essential expression of human identity. Braverman's argument is that capitalism requires the unity of conception and execution to be dissolved. Removing the option of conception from workers dismembers and degrades their human identity but this loss means nothing to the capitalist who now has more control over the worker. As such Taylor's ‘scientific’ prescription is not the best way to do work, but the best way to control an alienated workforce. Braverman concludes that ‘the accumulation of wealth which takes place at one pole of society in the capitalist system is, therefore, matched at the other pole by the accumulation of misery.’

Craft workers in the past and in artisanal workshops today do not face this alienation. They break their work down into tasks, some of which require their utmost skill and some of which are routine. However, their division of tasks does not require any loss in their power of conception. By contrast, while carrying out the simpler tasks, their creativity is released for wider conceptualization of the work. In contrast with large assembly lines, in small studios the need for incentives and control is minimal and the artisan is, therefore, free to pursue a wider and more balanced efficiency. Rather than the single objective of expanding each unit of capital, the craft-worker can instead aim at a personal balance between productive speed and satisfaction in the task at hand. Furthermore, the elimination of choice required by Taylor's principles applies not just to jobs but also to products. In many cases the individualized products of the craft-worker, while more expensive, hold an increased sense of value for their owners or users precisely because of the closer integration of conceptualization and execution that led to their creation. Our traditions of heritage, culture and art depend on the variety of regional or local solutions to design and production. However, the success of manufacturing in the twentieth century has led to a loss of variety, in modes of transport, in clothing, in building design and in decoration.

It would be unhelpful to over-romanticize the craft-worker, whose output rate is low and could not match the industrialized economies of scale in providing essential goods to a large population. However, the fundamental challenge raised by Braverman's analysis is a recognition that human labour produces a surplus, and it is a societal choice how much surplus is beneficial and how it should be distributed. At present, the capitalist solution has become dominant to the point of being almost unquestioned in the developed economies. Yet the polarization between the high satisfaction of those few who own capital and the degradation of the controlled many who do not is not the only possible societal organization. In pursuit of Industry 1.61803, there are opportunities to examine different solutions to the design of work, to production and to the distribution of the surplus of labour. It is likely that some of these solutions could lead both to a reduced material demand and to an increased satisfaction for a majority of workers with jobs redesigned to have at least some of the integration inherent to craft work. Aligning shareholder interest with workers interests, rather than the reverse alignment as often discussed, appears to be a fruitful area for new development.

### (b) Individuals and businesses: leisure

John Maynard Keynes, reflecting on this in his essay ‘Economic possibilities for our grandchildren’, predicted in 1931 that ‘for the first time since his creation man will be faced with his real, his permanent problem: how to occupy the leisure, which science and compound interest will have won for him, to live wisely and agreeably and well’ [29]. Keynes anticipated working hours reducing initially to 15 h per person per week, and then falling further.

Keynes’ observation arose from the assumption that human beings had some finite set of ‘needs’ that could be sated. In fact, as described in the brief history of manufacturing above, once these ‘needs’ were generally met in the developed economies by around the 1950s, manufacturers turned instead to creating ‘wants.’ If the objective of industry is that every unit of capital must expand, then demand must be expanded in order to absorb the output of ever more efficient production. The efficiencies of the economies of scale must, therefore, be matched by marketing campaigns to induce new purchasing. In contrast with Henry Ford's steady refinement of the Model-T, General Motors introduced the idea of a yearly model change in the 1920s, and manufacturers have since looked for innovations in form and function so that no purchaser's needs would ever be sated. Once a worker has earned enough to purchase the goods required to meet some basic set of needs, they can be induced to continue working and purchasing, to replace the first set of goods with a newer set with more features or the latest style. Without doubt some of these innovations have been beneficial, but, equally, many innovations aiming at some form of ‘convenience’ could only be sold with an induced demand. Gutowski *et al*. [30] describe the difficulty of developing re-manufacturing systems from consumer goods due to the increasing complexity of designs for the same basic function. Pencil sharpeners, toothbrushes, hairbrushes and bicycles are all now sold with electric motors attached for a ‘convenience’ that offers relatively little benefit over their previously un-motorized predecessors.

**Figure 3.**
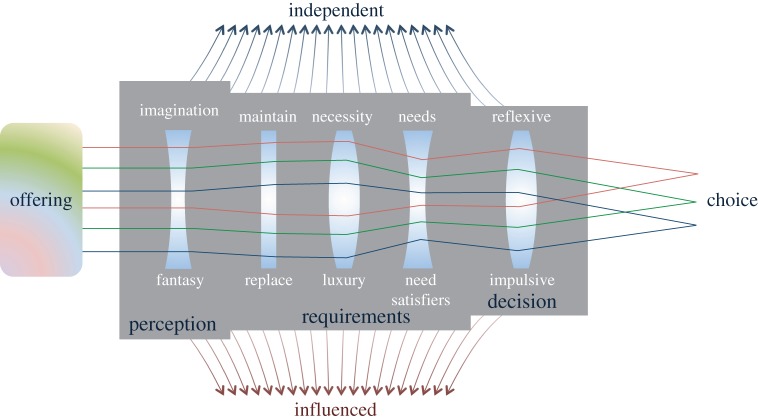
A representation of the insights into purchasing decisions revealed in this special issue. The figure shows how the offering of a product or service is filtered through several ‘lenses’ which determine consumer choice, and how these lenses may be influenced by marketing away from the outcome of a more independent choice. (Online version in colour.)

Persuading purchasers that they should be dis-satisfied with their existing goods so must ‘upgrade’ to the latest design depends on a control of perception parallel to the control over workers described in the previous section. Figure 3 is a representation of purchasing decisions arising from the papers in this special issue. The figure demonstrates how consumer perceptions of a product offering are filtered through several lenses before leading to a purchasing decision. The effect of each lens can be influenced by marketing activities away from what would have been the individual's more independent preference. Considering these lenses from right to left, Marteau [31] describes a ‘dual process model’ of decision-making, in which reflexive decision-making based on values and evidence is often trumped by impulsive or automatic responses. It is these impulsive responses which are the target of marketing activities aiming to override considered judgement about needs, with a quick acting reflex. Gough [32] presents a theory of human need that draws a distinction between universal, objective needs and subjective, socially determined ‘need-satisfiers’. He defines these need-satisfiers as the goods, services, activities and relationships that contribute to need satisfaction in any particular context. The intention of this distinction with needs is to avoid a paternalistic recommendation about which need-satisfiers are ‘necessary’ and which are ‘superfluous.’ Gough continues to discuss a dilemma which arises from this separation: if a policy on consumption motivates a change from high to low carbon emitting purchasing, will this reduce or worsen social inequality? This motivates a discussion of the difference between ‘necessities’ and ‘luxuries’. Quoting work by Druckman & Jackson [33], Gough finds that emissions associated with UK consumption would be reduced by just over a third should the entire population consume according to a ‘decent life budget’ determined by focus groups to meet an acceptable minimum standard of living.

Most goods, with the exception of infrastructure, are discarded and replaced before they are broken following either changed user needs or the persuasive influence of marketing campaigns about the benefits of a newer form of the same goods. Serrenho & Allwood [34] introduce the idea of ‘stock demographics’ to characterize the current stock of goods in a developed economy required to provide some level of service to a population. This allows analysis of the trade-off between maintaining or replacing goods and shows how innovations permeate through the total stock of goods in use. A broad literature reviewed by Skelton & Allwood [35] examines the environmental implications of replacing goods that become more efficient over time due to technical innovation. They find that office blocks and washing machines for example are typically replaced prematurely, whereas cars and planes tend to be kept beyond their environmentally optimal lifespans. Cooper *et al*. [36] in examining the longevity of steel intensive products identify four reasons for early replacement, and suggest alternative design strategies to allow an extended use of the material*.* However, despite the technical possibility of design and maintenance for longevity, marketing strategies are successful in persuading consumers to replace goods earlier than necessary, and as a result Roberts *et al.* [37] examine the causes behind such regretted purchases.

The final lens in figure 3 describes a much broader category of perception along a spectrum from imagination to fantasy. This distinction, introduced in this volume by Davison [38], recognizes that no product exists independently of its associations and meaning. Studying Coleridge and Murdoch, Davison [38] proposes that scientific facts about climate change are received through acts of either imagination or fantasy and that subsequent actions depend on these perceptions. Murdoch writes ‘The world which we confront is not just a world of “facts” but a world upon which our imagination has already worked.’ An imaginative act demonstrates a willingness to change one's image of the world through what one encounters and is required to step out of the cycle of ever accelerating production. By contrast, an act of fantasy involves a closing in and a projection of the world as one wishes it were—the outlook attributed to climate scepticism/denial and techno-optimism about supply-side solutions*.*

**Figure 4.**
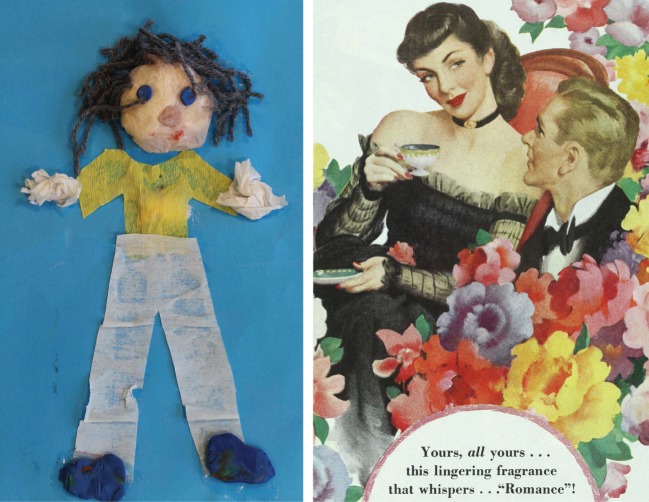
Imagination and fantasy: (*a*) a representation of the first author by his son, aged 4 at the time, whose interpretation depends on the positive benefits of imagination; (*b*) an advertisement for perfume in 1894 [39] (the contemporary equivalent would be more sexually explicit but have the same intent) convening the fantasy that purchasing the perfume will influence the attractiveness of the giver or user to each other. (Online version in colour.)

Figure 4 provides an illustration of this distinction between imagination and fantasy. The first image is an expression of imagination: the image bears little similarity to its target, but both to the creator at the time and to the recipient years later it carries a rich association of time, place, optimism and relationship. As Fletcher says [40], it ‘pays attention, it involves agency and it is fundamentally integrative.’ The second image, created by a marketing agency, conveys only fantasy. The viewer is given little space to add personal interpretation to the message that purchasing this particular perfume will make the wearer (or giver) more attractive to the other. This is a highly constructed message, strongly influenced by the manufacturer in the hope that the fantasy being offered in the image will attract sales. An independent assessment of the same product could only be made by smelling it in comparison with other offerings and making a selection based on the pleasure of the smell rather than the fantasy created by this and other images.

Keynes' forecast has so far turned out to be wrong because, whether by human nature or whether by the insidious marketing campaigns of profit hungry manufacturers, the satisfaction of our basic needs turns out to be insufficiently satisfying. Instead we inherently are or we are induced to be competitive, seeking to raise ourselves in the ranking of our peers, and this drives us to continue working hard for more income to buy more goods.

**Figure 5.**
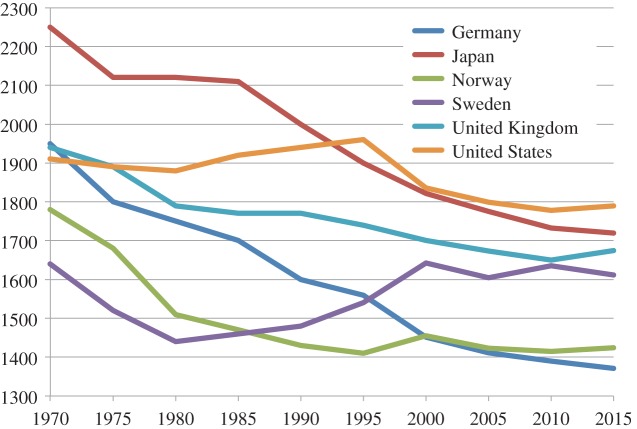
Working hours per person in employment for selected OECD countries, 1970–2015 [41].

Figure 5 presents data related to this story for several developed economies. Working hours have fallen for all the selected countries over the period 1970–2015 although, with the exception of Japan, the rate of reduction has been lower since the millennium. Apparently, therefore, leisure hours have increased albeit by not as much as Keynes forecast. However, these figures which are averaged over the number of people in employment need some careful interpretation. Devetter & Rousseau [42] note that the approximate stabilization of working hours in many OECD countries since the 1980s has been accompanied by an increase in employment levels, leading to an increase in total working hours for households. Thus, the unpaid work previously performed in households has become part of what Braverman calls the ‘universal market’ and he raises a concern about this. If services that were previously provided within households are now purchased in a market, they are potentially subject to the same degradation as other jobs controlled by capitalists, so less rewarding than previously. The reduced working hours per person in figure 5, therefore, do not necessarily translate into an increase in leisure and duly there is evidence that many workers would prefer to trade some income for more leisure time. Wheatley [43] examining this question among senior workers in UK industry found that around one third of private sector managers and public sector professionals were working more than the EU maximum working week of 48 h and the professionals in particular had a strong preference for reducing hours for less pay. More broadly, Erdogan [44] found that 90% of full time employees claimed that they would like to spend more time with their family and Campbell [45], therefore, comments that there is some doubt about labour supply theory in neo-classical economics if so many employees express a preference for different hours.

One particular concern raised in the literature around working hours is whether those working for longer, and typically earning more as a result, then spend their increased income on higher impact consumption. Vringer & Blok [46] use a physical basis for calculating the embodied or indirect emissions of household purchasing, to demonstrate that as household expenditure rises, apart from spending on petrol (which reaches a plateau) and food (which increases less steeply after some threshold) the energy inputs associated with spending increase very nearly linearly. Similar as yet tentative evidence [47] suggests that the household's environmental footprint increases with working hours and that longer working hours lead to more energy intensive, conspicuous expenditure, for example, on clothing, appliances, housing and travel [42].

Keynes' assumption that we would work less and instead focus on our best use of leisure proved to be wrong (although with increased life expectancy we do enjoy increased leisure in longer retirement) because we compete for social ranking by the purchasing made possible by increased income. We have, therefore, accepted commercial and policy frameworks predicated on targets of income growth and full employment. Yet this section has demonstrated that the pursuit of these targets is not necessarily guaranteed to achieve an optimum well-being, either in work or leisure. Other trajectories could be possible. For example, corporate directors today are legally required to take decisions that maximize the return to shareholders, that nurture each unit of capital to the maximum. An alternative legal framework in which directors were legally required to maximize the long-term welfare of their employees, would require a longer-term view of corporate health and broaden the management actions by which welfare could be promoted.

### (c) Individuals: ownership and usership

The commoditized products created by the economies of scale in manufacturing and marketed with constructed fantasies create little if any emotional connection for their owners. The most efficient designs for production often connect inaccessible sealed modules within products. The cost of assembly has been reduced so effectively that for many products the costs of maintenance, repair or upgrade exceed the costs of disposal and replacement. Yet this impersonal disconnection is not the only possible relationship between people and products. Our museums and heritage buildings tell a different story, of intrinsic value in objects themselves, linked to the stories of how they were made, the context in which they were commissioned and the story of their use over time. In an ethnographic study of the Eveny and Iñapiaq people, Bodenhorn & Ulturgasheva [48] cast light on their vital reliance on materials to adapt to extreme weather events in the Arctic. On the one hand, Bodenhorn and Ulturgasheva emphasize the need for flexibility in adaptation and stress the need for lightweight, mobile, multi-purpose possessions. Quoting Briggs, they note the experimental engagement with the material world by Inuit communities who tend to ask not what a new material is for but instead to speculate about how else it might be used. On the other hand, they point to the material needs of the Eveny people whose Soviet-funded more permanent settlements are now both under-resourced and inadequate in their choice of materials to respond to conditions affected by Climate Change.

**Figure 6.**
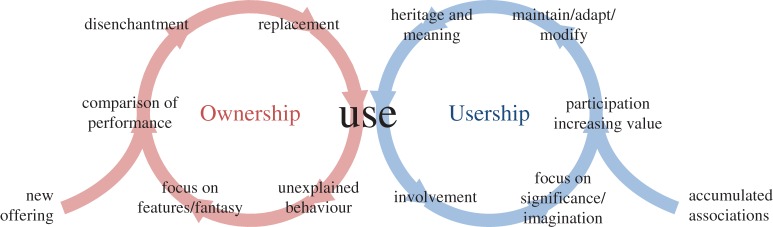
Contrasting the patterns of ‘Ownership’ and ‘Usership’ from Fletcher [40]. (Online version in colour.)

Fletcher [49] reports evidence gathered from a multi-year study of the way people use garments, ‘the craft of use’. Fletcher [40] develops this idea into a contrast between ‘Ownership’ and ‘usership’ summarized in figure 6. ‘Owners’ typically unable to explain the behaviour of a product or personalize it, focus on its performance and its delivery of the fantasy (as discussed in [38]) associated with it by marketing. Inevitably their experience becomes disappointing as the fantasy fails to deliver, so over time they compare its performance with newer offerings. They become disenchanted and purchase a replacement product long before the original has expired in the hope created by the next fantasy. By contrast, ‘users’ are involved in their products. They care for, maintain and keep using the original and build up positive imaginative associations with it, linked to a relationship with the product built over time. Their participation in using the product increases its value over time. Rather than comparing the product's performance with more recent offerings their experience is of growing heritage and meaning.

Fletcher's evidence is found in garments and her book includes nearly 500 stories of garments that over time have developed a personal significance for their users. In a preliminary exploratory study of a different sector her work has led to a study of the ‘Usership’ of Land Rover Defenders [50]. These vehicles which were designed to be easy to repair and modify have created a strong user community and around 80% of all Defenders ever made are still on the road. The exploratory study suggests that the relative simplicity of the design draws users in, enticing those without previous mechanical experience to develop skills in repair and in time to modify and customize their vehicles.

‘Usership’ as described by figure 6 may be more fulfilling than ‘Ownership’ and is likely to lead to a reduced demand for new materials as existing goods are nurtured and, with accumulated experience, grow in value to their user. The success of a strategy to promote ‘usership’ may depend on the time budgets of owners, on whether they are prepared to invest time in the relative inconvenience of maintaining a more personal product, rather the replacing a more anonymous one. As an example of one approach to addressing this inconvenience, Frenken explores three scenarios of a ‘sharing economy’ revealing that the potential winners and losers from sharing, as a strategy of ‘usership’, vary depending on how sharing is established [51]. At present, however, the corporate influences of figure 3 generally pull individuals against any idea of usership. Despite an optimistic academic literature about ‘product service systems’ [52], ‘frugal innovation’ [53] or ‘servitization’, evidence to date suggests that incumbent businesses have not found profitable opportunities from the idea of ‘usership’ and the ‘servitization’ of developed economies has not reduced their material demand but merely exported it. In contrast with this hypothetical optimism, Gutowski *et al*. [54] anticipate that global requirements for materials may only ‘saturate’ (reach a level where most purchasing is for replacement goods rather than to serve new entrants) at around double today's stock levels. However, the evidence of the preliminary study of the Land Rover Defender suggests that products could be designed to support and encourage user engagement. Gutowski *et al.* [30] provide an important caveat about the opportunity for such engagement in his evidence that re-manufacturing is inhibited by increasing technical complexity in many goods. However, for many products, the opportunity remains open for individuals to choose a path of ‘usership’ and Fletcher's evidence suggests that for some people at least, this may be more satisfying.

We cannot imagine discarding our heritage buildings, the treasures of our museums, or the unique objects passed to us from our grandparents, because as much as their design we recognize and appreciate the human endeavour that created them. The cover image of this special issue is a Kintsugi bowl, repaired by ‘golden joinery’ after breakage in such a way that it gains a new beauty. The story conveyed by the repair, encompassing its life before and after damage, adds to its value. Signs of use or wear augment rather than diminish the bowl's aesthetic [55]. However, the economies of scale, which eliminate human expression in work simultaneously eliminate that sense of endeavour and lead to the production of goods with which we sense very little connection. Partly as a result of this Roberts *et al*. [37], in examining instances where consumers regret their purchases, draw attention to mass-produced electrical goods and discuss the loss of well-being associated with their regret.

Xenos [56] presents an account of the history of austerity in the Western tradition. Despite the overarching trend towards ‘more’, there have always been those who have argued for ‘less’, from Socrates and the Stoics to Pope Francis and Marie Kondo. The socio-political connotations of the austere life have differed greatly from the use of austerity by various religions to recommend themselves to ‘the common people’ to whom, according to Adam Smith, ‘the vices of levity are always ruinous,’ to the elitist undercurrent associated with bankers ditching their stuff as part of the new craze for minimalism, and from Ambrose's assault on greed in pursuit of cohesion within the church to Jerome's use of austerity as a badge of distinction from ordinary, lesser Christians. Xenos argues against the current appropriation of the term ‘austerity’ to the field of economics. Based on the etymological ‘bitter’ roots of the word, the common use of the term ‘austerity’ in the economic context does not take into account the fuller, historically rooted and more virtuous account of austerity as an aversion of ostentation and excess. Xenos argues that economic policies labelled as ‘austerity measures’ are in fact based on an ascetic programme of self-restraint and deferred gratification (for future growth) aimed at the weakest in society. Opponents of this logic propose economic stimulus in pursuit of growth. Xenos argues that, in contrast with both the use of pro-austerity and anti-austerity within the current policy dialogue, a truly austere politics would require the abandonment of the shared logic of growth. Both Xenos [56] and Kallis [57] warn of the potential hidden environmental burden that can be instigated by ostensibly pro-environmental, dematerializing measures. Xenos, quoting Chayka writes ‘this new minimalist lifestyle always seems to end with enabling new modes of consumption—a veritable excess of less’ and Kallis points to the risk that more fossil fuels could be expended in the pursuit of decarbonization.

### (d) Individuals and government: the common good

**Figure 7.**
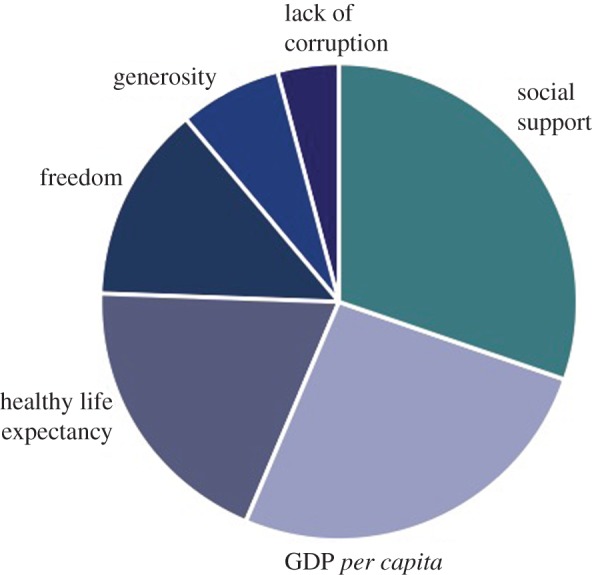
A decomposition of the determinants of happiness across several countries [59]. (Online version in colour.)

Recent government policy proposals have been filtered according to their impact on ‘growth, jobs and bills’ yet as cited above, 90% of employees stated that they would prefer to spend more time with their families [44]. In a different challenge to these universal policy evaluation criteria, Kasser [58] reviews literature on the correlation between pro-ecological behaviours and well-being and finds evidence of a self-reinforcing ‘virtuous circle’: pro-ecological behaviours lead to greater life satisfaction while more contented people are more likely to engage in such behaviours. Consistent with this, Whitmarsh *et al*. [59] finds dematerialization behaviours to be intrinsically rewarding and fulfilling for individuals. Extending this idea from the individual to wider society, a body of work has developed to examine alternatives to GDP as a metric of well-being, prominent among which is the idea of ‘happiness’ [60]. The use of a self-reported metric of happiness is still open to much challenge, but the analysis of data gathered in this area over many years has produced two tentative conclusions: beyond some level, happiness grows much more slowly if at all as income increases [44,61]; the drivers of happiness, broken down in figure 7, include GDP *per capita*, social support (having someone to count on in times of trouble), healthy life expectancy and freedom to make choices [62].

Alfred Marshall, one of the pioneers of neo-classical economics defined four categories of human welfare—social, religious, political and economic—and asserted that economics is concerned only with the economic component [63]. To some extent this confirms the decomposition of figure 7, although the figure includes no direct attribution to religious welfare. However, the importance of Marshall's original constraint on the role of economics is to provide a further challenge to the primacy of economics in political decision-making. If Marshall is right, economic consideration of policy options should be balanced by social, religious and political evaluation, yet this is not the case in the Western democracies today.

Increased mobility, rapid developments in information technology and the extraordinary power of commercial and media influences have diminished the importance ascribed to local community. In mediaeval times in the UK and in rural regions of developing countries today, location and rootedness were essential features of life. Local collective decision-making was a vital component of any national political system. Without such rootedness, the definition of community has become uncertain and so the possibility of a collective pursuit of a ‘common good’ has been reduced. Justice, social welfare and healthcare are seen as the responsibility of remote decision-makers, ‘them’, and there is little if any collective local choice by ‘us’ about public spending priorities. Schumacher proposed in 1971 that a more localized system of production would create a natural feedback from consumers to producers, to ensure social and environmental responsibility in industrial systems [64]. Instead, the rush to globalized industry has further disconnected consumers and producers, but, in parallel, the systems of social welfare have become as remote as manufacturing.

Xenos’ paper presents a challenge to return decision-making from ‘them’ to ‘us’ through a renewed focus on ‘civics’ and the collective definition and pursuit of a common good. Whitmarsh *et al.* [59] examines how the specificities of culture coupled with social psychological processes could support material demand reduction or not, and concludes that Brazilians, for example, are more likely to reduce their material demand than the British. In the comfort and wealth of the developed economies since 1950, political engagement has generally declined, and the single metric of GDP growth has by default become the primary target of political decision-making. A tentative conclusion of this section is that a wider reconnection of people to politics, perhaps at more localized scales, could question this single measure of ‘the common good’ and lead to a broader set of goals for political action.

### (e) Government: challenges to conventional economics

In most developed economies, policy decisions are informed above all by the models of neo-classical economics. Aidt *et al*. [65] describe how the argument for material demand reduction is viewed in this framework. They explain that a carbon price can be an effective policy instrument in the eyes of neo-classical economics as it offers flexibility over how emissions savings are achieved, it targets the source of the underlying problem directly and in so doing it reduces the risk of unintended consequences. This argument is very widely accepted in the national and international policy communities of the rich nations and for around three decades the neo-classical proposal that climate mitigation should be implemented via a tax on carbon emissions has stood prominent above all others. Unlike any other measure (regulation, incentives, information, bans) the proponents of the carbon tax argue that it does not ‘pick winners’ but will instead force a search for the lowest cost solution.

The primary challenge to this widely held and theoretically complete solution is that in three decades of international negotiation, we have made very little progress in agreeing such a tax at global scale. Where local schemes, such as the EU emissions trading scheme have been developed, then despite efforts to address the over-allocation of credits that undermined earlier phases of the scheme, the carbon price has been forced down to a very low level, due to a range of factors, most importantly the unforeseen effects of the 2008 financial crisis [66]. The scheme has, therefore, had little if any effect on total emissions. Victor [67] argues that this is inevitable and that an all-encompassing international agreement simply cannot be negotiated for an issue of this complexity.

**Figure 8.**
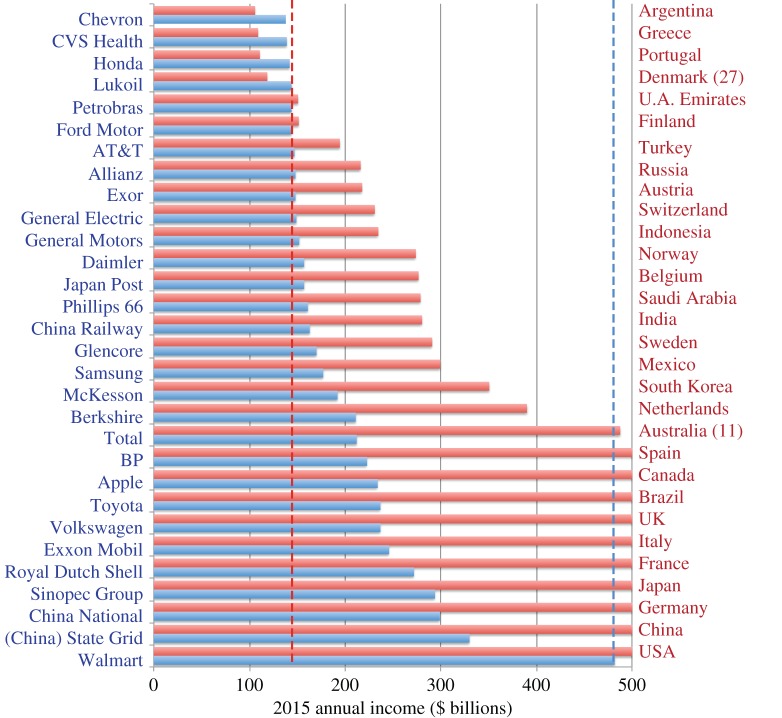
The largest 30 countries and corporations ranked by income. The *x*-axis is common to both series to underline the similarity in the magnitude of corporate and national incomes [70]. (Online version in colour.)

**Figure 9.**
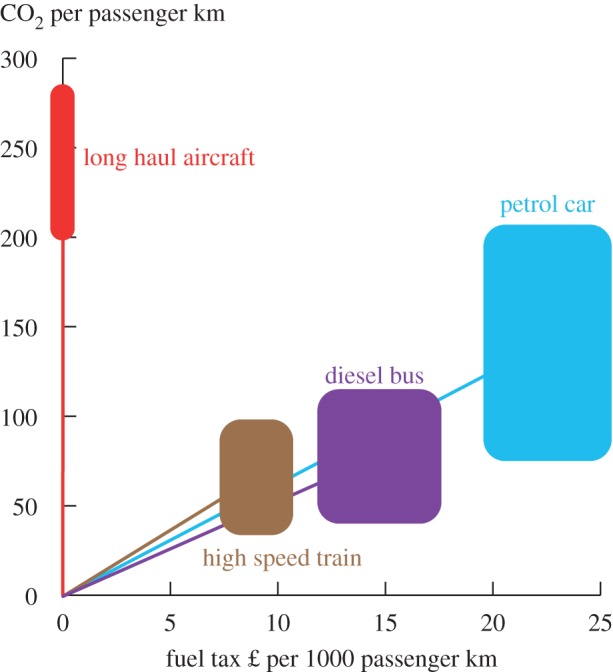
Fuel tax in the UK and the emissions of different modes of transport ([71–73], emissions figures from IPCC, [74], fig. 8.6).

**Figure 10.**
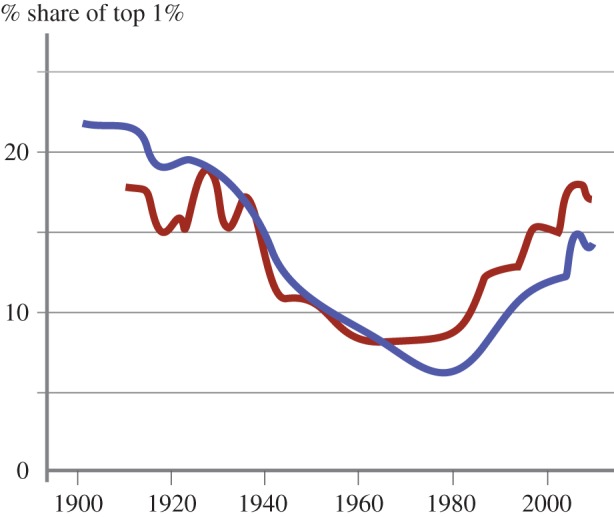
The fraction of income earned by the top 1% of earners in the UK (blue) and USA (red) has expanded dramatically since 1980 (reconstructed from [76]).

However, in addition to this fundamental problem of negotiation, this special issue contains several other challenges to the conventional wisdom of neo-classical economics:

— The neo-classical economic framework described by Aidt *et al*. sees consumers maximizing utility subject to their budget constraints and producers simultaneously maximizing profit subject to costs. Therefore, no-one is motivated to reduce demand—i.e. to opt not to meet demand from customers at prices that are profitable to producers. For neo-classical economists, preferences are ‘revealed’ through purchasing behaviour. You wouldn't buy it if you didn't want it, so if you bought it you want it, and don't want not to have it. Contrary to this, Marteau [31] sees much of demand governed by non-conscious processes. Preferences are then only being revealed to the extent that they are reflected in non-conscious processes. Whether or not you ‘want’ demand reduction suggests a conscious process, which cannot necessarily be ‘revealed’ by demand behaviour alone. Marteau, therefore, suggests that it is not necessarily the case that no one wants material demand reduction, just that we might not be thinking about it.
— Skelton [68] examines the effect that a carbon tax on energy would have in incentivizing material demand reduction taking into account the sequential nature of decision-making along supply chains. She explains that a carbon price will only incentivise material demand reduction in downstream sectors (such as the car industry or the construction sector) to the extent that it is ‘passed-on’ in the form of increased energy and subsequently increased steel prices, and explores different situations in which such pass through may be inefficient. Of the arguments considered, compensation mechanisms that seek to address the risk of carbon leakage are likely to act as the greatest hindrance to material demand reduction: unless upstream companies are encouraged to make windfall profits from these types of schemes, downstream incentives for reducing demand for embodied emissions intensive materials will be weakened. This paper explains how the difficulties in implementing a global carbon price described by Victor [67] can to some extent be overcome through unilateral action coupled with compensation schemes but translate into weaker incentives for material demand reduction.
— Kallis [57] makes a different challenge to the current application of economics in driving government policy by observing that what economists frequently describe as ‘externalities’ are in fact cost-shifting successes. In contrast with the theoretical ideal of a free market, Kallis quoting Kapp [69] notes how different players in a democracy have different power to exert influence over policy and hence the economists’ ‘externalities’ are not absolute functions of the neo-classical model, but a result of power-play and negotiation. Figure 8 underlines this point by showing the total income (tax revenue and turnover, respectively) of the top 30 countries and corporations in rank order. Walmart, the largest corporation by turnover is larger than every country below Australia (which has the 11th largest tax revenue). All the corporations shown have higher turnover than the 27th country (Denmark). The large size of these corporations gives their (unelected) directors significant influence over (elected) politicians, part of which can be used to externalize costs that must then be borne by society.
— One topical example of this is the negotiation by the energy intensive industries in Europe to be exempted or compensated from carbon charges. Figure 9 illustrates a different example related to fuel tax. Within the UK, fuel tax on road vehicles and trains has been applied to give a relatively consistent charge for the emissions intensity of different modes of transport. However, aircraft are currently exempt from fuel taxes, so the mode of transport with the highest emissions per passenger kilometre (and with even higher global warming potential due to their release of NOx emissions which modify ozone concentrations) has externalized all the costs of its impact on global warming.
— An increasingly vocal group of economists are questioning the validity of the use of GDP growth as a policy target. Authors such as Picketty [75] have drawn attention to the dramatic increase in inequality that has occurred since the 1980s in the USA and UK, in particular. Figure 10 illustrates this, showing how the top 1% of earners in these countries now take 15–17% of all income, up from 5 to 7% in 1980. The bottom 90% of earners, the bulk of voters in the UK, have seen no real income growth in the past 30 years so the aggregated and scalar target of GDP growth is clearly failing to lead to a benefit for most of the population. Aidt *et al*. [65], therefore, argue that government policy including mitigation strategies should be tested for its distributional consequences as well as for its impact on total GDP.
— Kallis [57] takes an even bolder step, and anticipates that GDP growth will prove to be incompatible with material and energy demand reduction, so therefore recommends that we expect that policy actions to reduce greenhouse gas emissions will reduce GDP. He argues that the key driver of growth since the Industrial Revolution has been the substitution of energy for labour. The conventional economic argument that this driver is a substitution of capital for labour fails to acknowledge the energy inputs required by productive industrial equipment. As a result, using less energy means a reverse substitution back to less productive labour causing prices to rise (as externalities are internalized) and thus potentially destabilizing society. By viewing the economy as fundamentally material, Kallis sees de-growth as an inevitable by-product of constraining the use of highly productive energy. The key challenge then is the social stability of a transition to a smaller, carbon-constrained economy. This remains a postulate rather than a proof at present, but will prove to be true unless it is possible to define industries that scale (measured in sales per person) without an equivalent scaling of energy inputs. Many service industries such as cleaning, hair dressing or the creative arts show no such scaling. Techno-optimists draw attention to the rapid scaling of the so-called ‘tech’ industries, providing services through software on mobile devices or via the Internet, as a potential low-energy form of growth. However, this can scale nationally only if the beneficiaries of the growth spend their income primarily on purchasing such services, rather than purchasing physical goods or energy services. To date, as Druckman & Jackson [77] show, income has been the key predictor of personal carbon footprint, because richer people travel more, have larger homes and purchase more goods. The notion of a scalable service-based and low-energy industry, therefore, remains unproved, hence Kallis' forecast that de-growth will be a prerequisite for energy demand reduction.

Outside of these challenges which are raised in the papers in this special issue, the discussion meeting raised three further questions about the application of economics in political decision-making today.

**Figure 11.**
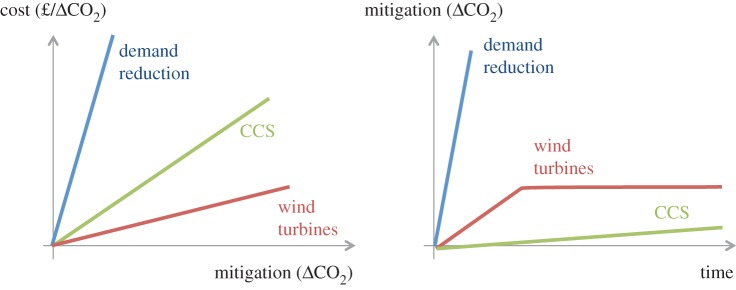
A qualitative representation of the marginal cost of deploying various climate change mitigation measures, demonstrating how the cheapest option may have limited effect either due to its total potential, or due to the maximum rate at which can be deployed, neither of which effects are ‘visible’ within the marginal analysis of neo-classical economics. (Online version in colour.)

— The coefficients of models used by neo-classical economists are determined from observation of small marginal changes about the present state, which is assumed to be somewhere near a notional ‘equilibrium.’ However, the change required to mitigate global warming is not marginal but radical and in this case the model is likely to be nonlinear and the coefficients are unlikely to be useful predictors: the most efficient marginal choice may not scale sufficiently to reach target levels of mitigation, in which case pursuing only the cheapest option simply delays the implementation of other options which do scale. This is illustrated in figure 11 which shows (qualitatively) that deploying wind turbines and CCS in the UK are apparently cheap options, but cannot scale to achieve target levels of emission reduction. Meanwhile, retrofitting buildings to reduce their heating and cooling requirements and redesigning cars to be fit for a low carbon world is apparently more expensive, so will never be chosen by a marginal analysis, but these two options have far greater total potential. Similarly, deploying CCS appears to be relatively cheap, but the rate at which it can be implemented is very much slower than the rate at which the population could change their travel preferences in a crisis. The cost-optimizing models which are used in policy circles to evaluate new investments in the power sector are, therefore, probably inappropriate for informing decisions about mitigation.
— The definition of ‘cost’ is crucial to understanding the challenge of implementing mitigation options yet cost is rarely defined with sufficient precision. ‘Cost’ may refer to the investment cost (for a private sector firm investing in a new power station), to a government intervention (to motivate the private sector firm to invest differently than it would if solely motivated by its own return) or to the compensation that must be offered to individuals to persuade them to prefer a lower emitting behaviour or product (such as a smaller, slower car). The definition of ‘cost’ also depends on who wins and who loses from some change to current practice. The cost to the airline industry of imposing a fuel tax in line with that faced by road users would be a benefit to tax payers and would stimulate consumer demand for other services including alternative modes of transport. The discussion of mitigation in policy to date, exemplified by the 5th Assessment report of the Intergovernmental Panel on Climate Change [74] has assumed that ‘cost’ has a single definition and describes a loss. This is not a complete picture as ‘cost’ describes a transfer of funding from one activity to another. Understanding options for reducing energy and material demand requires metrics other than investment cost and this area merits significant further investigation and clarity.
— The concept that ‘labour’, ‘energy’ and ‘capital’ are substitutable factors of production depends on the assumption that the goal of production is to maximize returns on capital. However, if the purpose of production is to augment human lives, then as Braverman [27] points out, this concept is absurd. The separation of conception from execution which is essential to allow the thought process that all three ‘factors’ are in some way equivalent, fundamentally denies humankinds' unique attributes, yet these do not currently feature in economic analysis.

The neo-classical model of economics is self-consistent, so within its own assumptions, it is ‘correct’ and defensible. However, the ideas in this section question whether the model is ‘fit for purpose’ as the main assessment of government policy proposals for mitigation. Many of the questions raised here lead towards a research agenda and an opportunity for economists to find a new basis for informing government policy discussions with absolute clarity about the assumptions behind their predictions. For the moment, however, a conclusion from the challenges listed above is that it is essential to inform ministers and the public of the wider implications of their choices. Two priorities are to raise awareness of how corporate actions achieve cost externalization and to promote policies that link mitigation with the aim to reverse the increase in inequality that occurred during the past thirty years' pursuit of GDP growth.

### (f) Businesses and government: lock-in and the timing of change

Investment decisions in fixed assets are made with an intended lifespan, over which the funding of the investment is written off. The decision to build a blast furnace, a bridge or a new school is based on a predicted lifespan. The funding to make the purchase will be predicated on revenue arising from the use of the asset over this time. Managers will be reluctant to abandon the asset during this period unless they can sell it, so during the intended lifespan are in effect ‘locked-in’ to using it as intensely as possible. This is both a practical action, to recoup the investment costs, but also has an emotional component: the managers who bid for funding to purchase the asset were successful in making the case for investment. Deciding on early replacement would require that they admit that their case was wrong. Policy decisions have some similarity to these investments. The ‘cost’ of implementing a new policy depends on the political capital that must be expended to have it accepted and the higher this cost, the longer the policy is likely to last and the more its proponents will defend it. During the period of lock-in, leaders committed to defending past decisions will be eager to subscribe to external ideologies that support their past decisions and this creates a miasma of ‘false hopes’ about mitigating climate change. Incumbent coal and gas businesses and their political supporters exaggerate the rate at which CCS can be deployed because it appears to justify their past investments in fossil-fuel power plants. Material intensive businesses invest large marketing budgets to reaffirm their commitment to a ‘circular economy’ by focusing on what might happen when their products are discarded, rather than by reducing the volume of material they cause to be made.

The consequence of this effect is that there are only brief windows during which large-scale change can be implemented, after which the change will be locked-in and defended. Change will occur when the previous investment (in assets, policy or branding) can be forgotten by investors or voters, when managers or ministers have their attention drawn to an opportunity and when an attractive alternative is available. The change requires all three of these conditions to occur at the same time and this occurs only in brief ‘windows’. This idea derives from an emerging body of work, largely in the USA, on ‘policy entrepreneurship’ [78] and is used to motivate the activities of such entrepreneurs—in business and politics alike—to be prepared with proposals at the time that window of acceptability and interest is open.

At a domestic scale, the same lock-in with limited windows of change influences the carbon footprint of households, most particularly when moving house [79]. The decision on house location locks its occupants into certain transport options and the size of the house locks them into commitments to purchasing carpets and furniture and to requirements for space heating that are difficult to change without moving house again. Patterns of behaviour are also established within these windows of change and become locked-in via habits and routines, which are highly resistant to change [80].

In the pursuit of Industry 1.61803 with greatly reduced material demand, the reality of lock-in and windows of change should inform the entrepreneurial actions required ahead of each window. During periods in which change is impossible, entrepreneurs who are already designing options for physical change must also be preparing the ground for decision-makers so that during the brief window of opportunity, they are empowered to implement change. This is nicely illustrated by the informal ‘Overton window’ [81] which describes a scale of political acceptability from ‘unthinkable’ to ‘popular.’ At present, the idea of material demand reduction would look ‘unthinkable’ but as public concern about climate change grows and the inefficacy of current supply-side measures becomes more apparent, it might become merely ‘radical’. With time and appropriate communication other positive messages could continue the changed perception so that demand reduction could move on to be ‘acceptable’, and eventually ‘sensible’ and ‘popular’. At worst, a crisis such as war or significant resource shortage, would lead to a rapid change in perception. For example, during the Second World War, rationing and material shortages were rapidly accepted in pursuit of a wider national goal. Less dramatically, the positive ideas gathered from this issue and summarized in this editorial could be part of this changed perception, as could a much wider range of social forces. Pope Francis' widely read and praised encyclical *Laudato Si* [82] placed the responsibility for mitigation firmly on the shoulders of richer consumers, focusing on personal behaviour rather than optimism about technological fixes. Alternatively, as Xenos [56] reports, fear of austerity as shortage could be transformed along the lines of Socrates view of austerity as something which combines virtue and justice. Tanya Harrod [83] also describes different perceptions of material, through art created from waste, or in Japanese tea ceremonies. Material demand reduction is ‘unthinkable’ in the Overton Window today, but the evidence of this special issue suggests that there are sufficient opportunities to gain experience of such reduction that policy entrepreneurs can use to build up the case for action next time a policy window combines with public interest in the area of material use and its impact.

### (g) Individuals, businesses and government: public agendas

**Figure 12.**
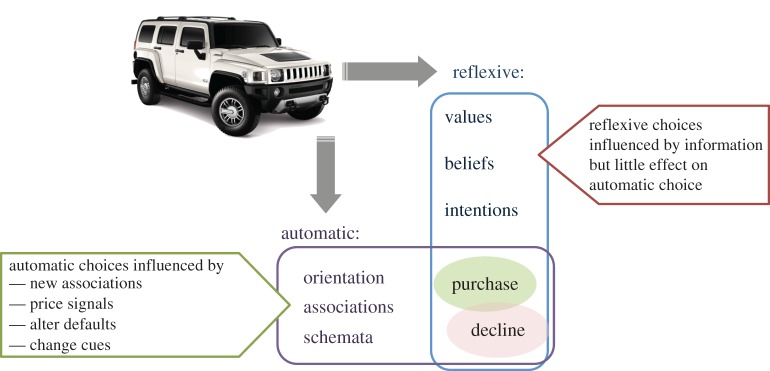
The ‘dual process’ model of human choice and possible policy interventions. (Online version in colour.)

The pursuit of options to reduce material demand, which is not currently the agenda of any major stakeholder group, has some parallels with the pursuit of public health. Marteau [31] reflects on the lessons that might be transferred from public health to demand reduction, starting with the most challenging statement that better information about negative consequences has a weak influence on future choice. Instead Marteau presents a ‘dual process’ model of choice illustrated in figure 12. This expands one of the axes in the earlier model of figure 3 to contrast the two processes of ‘automatic’ and ‘reflexive’ choice. Better information informs reflexive thinking but this has much less influence over purchasing decisions than less conscious ‘automatic’ reactions. The evidence reviewed by Marteau suggests two forms of intervention more likely to influence choice: changing the presentation of the product and changing its associations. The first change might include altering the defaults (for example offering a low-energy solution as the default option in purchasing a new service, and requiring a user action to change to a higher energy option) or changing the cues associated with a product, as happens when health warnings are made visible on cigarette packaging. The second change occurs when actions away from the product change its associations, for example by an information campaign intended to build negative perceptions of the environmental impacts of larger cars, or using price signals from taxation to adjust preferences.

Marteau's evidence on policy implementation around public health concerns shows, disappointingly, that campaigns based simply on information are most acceptable both with the public and in corporations, even though they are the least effective interventions. However, public acceptance of interventions increases if they are backed by compelling evidence of effectiveness, if the intervention focuses on benefits rather than risks and if the intervention is designed so that the ‘blame’ for needing the intervention is attributed to an external party, rather than the decision-maker themselves.

These actions to increase the acceptability of interventions all require channels of communication. Indeed the agenda for how societies talk, think and act is largely set by mass media and above all by television programming. Smith [84] explores this theme and starts by establishing the criteria that must be satisfied by a proposal for a new programme in order for it to be considered by broadcasters. These criteria generally include the need for a human subject with heroes and villains, a place where action happens, a story which is often aspirational and has a denouement and a presentation that has implications for households or individual characters. These are challenging requirements when exploring the need to mitigate climate change by reducing demand. Climate change is a threat, but not associated with any specific villain, location or moment in time. Responding to climate change requires collective action which denies the likely role of individual heroes. The action is global, and the ideal denouement is the absence of a problem in the future.

One of the contributions of this special issue is to suggest various forms of unexplored aspiration that could be pursued as part of an agenda of reduced material demand. The discussion around Smith's paper, triggered by his survey of previous presentations of demand in programmes, suggested some options for creating positive programming. Comedy has proved to be a vehicle for discussing issues around consumption, most notably in the UK with the BBC's ‘The Good Life’ programme in the 1970s contrasting a high-consumption couple with their neighbours pursuing an overtly ‘alternative’ lifestyle of self-sufficiency. Situation comedies (sitcoms) have been used as a vehicle for information sharing, most notably with the BBC Radio 4 series ‘The Archers’ being designed to share best practice in agriculture. Other possibilities include the idea of competitions (for example, in South Korea, LG and Samsung compete in challenges to repair domestic white goods), natural history style documentaries focusing on human behaviours and the exploration of increased and different forms of leisure.

The fact that information is not a strong driver of change is an important challenge to academics thinking about mitigating climate change as the urge to present better and clearer information is inherent to research training. Smith's paper therefore establishes a crucial opportunity for researchers relevant not just to television but to all media storytelling. It is hard to win long-form television coverage for most complex issues, but the multiplication of platforms and increasing influence of social media allows the demand reduction research community to pursue a strategy of experimentation with storylines and storytellers. Sufficient investment of time and imagination will ensure that some will reach or influence the still-dominant mass medium of television, and sharing these via social and other media platforms is in any case a ’no regrets’ route to increased public attention. Reducing demand for energy and materials is a fundamental necessity to mitigating climate change but only very rarely achieves public prominence, so is currently unlikely to motivate significant interest from ministers. In order to engage the political and wider policy community in the process of change in the process of change, demand reduction must, therefore, become a welcome part of the public agenda. As much as future research must aim to clarify the value of different options for initiating demand reduction, it must equally seek the stories and other avenues by which it can become compelling thread in media storytelling.

## 3. Who could do what, now?

This issue arises from an attempt to engage a broad range of academic disciplines in discussion about finding a transition to Industry 1.61803 and implementing material demand reduction. In attempting to build connections between so many subjects it inevitably raises important and compelling areas for future research. These will be examined in the next section but the challenge of mitigating climate change is not merely an academic problem. The need for action is urgent yet actions to date have not reduced the rate at which global emissions are increasing so the top priority from this issue is not further research but immediate action. Alongside other colleagues, Anderson [85] has been effective in drawing attention to the urgency of the problem, and in the spirit of his work, before asking about future research, we must address the higher priority question ‘who can do what, now?’

**Figure 13.**
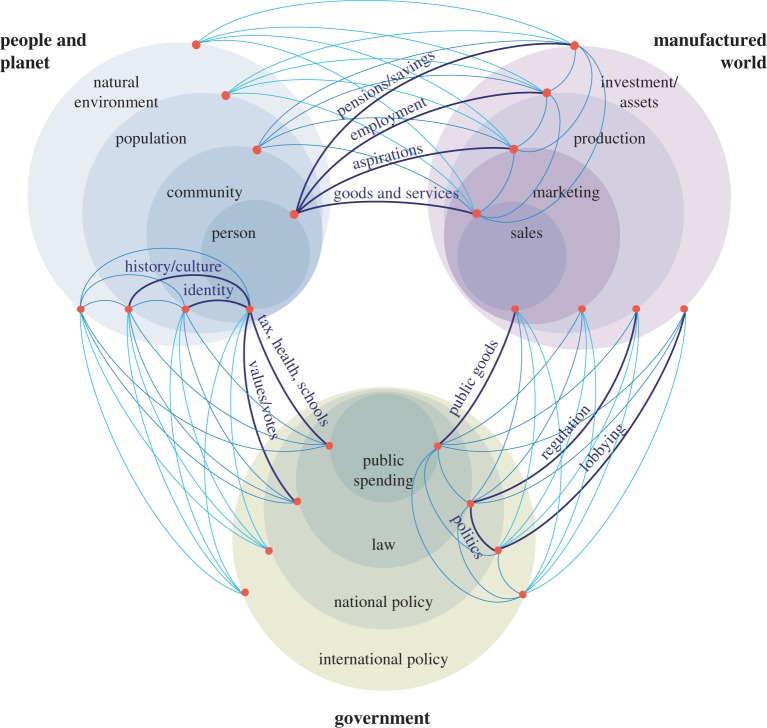
The three main stakeholder groups of figure 3, expanded into layers of increasing size, and with illustrative relationships among them, each of which could be influenced by either party. (Online version in colour.)

To attempt to develop an answer to this question, figure 13 presents the same three major stakeholder groups as used in figure 2, but now with each group shown in layers of decreasing immediacy. The central layer of each of the three groups, people, sales and public spending, determines the flow of money, energy and materials in the economy. Each occurs in a context of increasing scale and influence, but with slower rates of change. The figure illustrates the fact that at every level of scale, each grouping within any one of the three stakeholders has some relationship with each level of the other two. A simple answer to the question ‘who can do what to whom’ is therefore that either party in every one of the relationships in figure 13 could choose to act to influence the other towards reducing material demand. For example, communities developing a shared sense of values about material consumption could act as a lobby to influence national and international policy processes or, in reverse, policy could influence the choices open to the community. Equally, marketing departments which develop the messages that associate aspirations with particular products could promote alternative associations of heritage and conservation, or the individuals to whom the messages are directed could choose to contradict the marketeers' intentions by refusing to support material waste in their purchasing. At this rather abstract level, both at work and at home, every individual could act within the relationships in which they are engaged to create influence towards reduced material demand.

In an attempt to make this more specific, the seven insights arising from this meeting and presented in figure 2 suggest that:

— Manufacturers could re-examine their approach to job design and explore the extent to which restoring creativity and choice to their production workers enhances both the experience of their jobs and the perceived quality of their output. In parallel, journalists writing to inform consumers could contrast the experience of buying many ‘inhuman’ commodities against buying few products with the additional significance of craft skills applied in design and execution.
— Some individuals working long hours could explore options to work less and find increased satisfaction from using increased leisure in activities other than intense spending. Occasional government actions have sought to attract people from (unrewarding but high paid) work to secondary school teaching, recognizing benefit to both pupils and teachers from this transfer. Ministers in departments of work and business could offer additional support to similar schemes, such as Voluntary Service Overseas, to raise the profile of opportunities to create personal satisfaction through non-paid work at various levels of commitment.
— Innovation managers in corporations could explore the design of products aiming to facilitate usership with a broad examination of associated business models, while craft workers acting through community groups, writers, charity shops and technical colleges could expand their communication of the skills and rewards of engaging in the maintenance, repair and adaptation of existing products.
— Policy entrepreneurs from different sectors could map and evaluate the effectiveness of the widest range of interventions and instruments that might stimulate a reduction in material demand in preparation for the confluence of political and public interest that will grow as our experience of climate change increases and will lead to specific windows of opportunity when material issues become a public concern.
— Anyone involved in proposing or evaluating policy changes could focus on their implications for inequality instead of GDP growth. Any statement about the cost of a policy option could be described with sufficient detail to demonstrate how the option would lead to a transfer of funding from one source to another and to identify the winners and losers of this transfer.
— Community groups, the leaders of local governments and journalists working with local media could aim to stimulate a richer engagement of local opinion in local decisions, to enliven discussions of the common good at the level of villages, towns and cities. These discussions might also provide an opportunity to raise awareness of the intrinsic satisfaction and fulfilment arising from dematerialization behaviours.
— The programme development directors of media channels could through commission or competition seek new ideas for exploring the value of materials in attractive programming and meanwhile academics exploring climate mitigation could engage creative writers and others as part of their normal research output and engagement activity, to explore new opportunities for being present in the public agenda.

**Figure 14.**
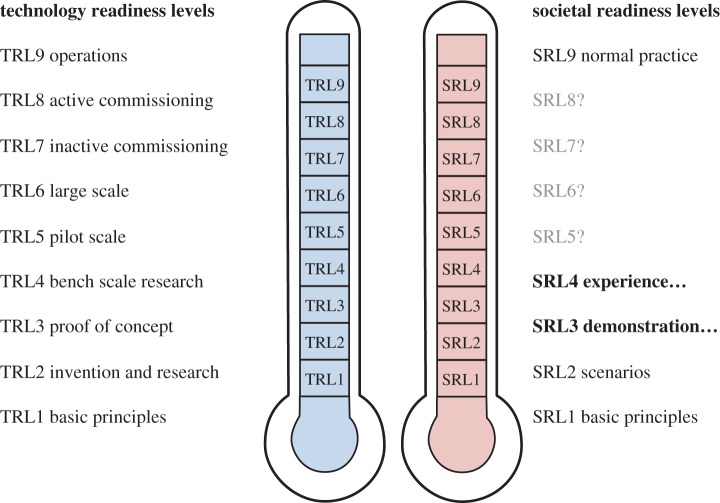
A proposed scale of ‘societal readiness levels’ to parallel Motorola's famous ‘technology readiness levels.’ (Online version in colour.)

Above all, a crucial requirement to pursuing the agenda of material demand reduction is to develop experience of living well with less material production. While the introduction of this paper set out the argument for why this is essential, the idea is counter-cultural and remains a largely theoretical possibility at present. Within the world of technology innovation, Motorola's scale of ‘technology readiness levels’ [86] is widely used as a basis for understanding innovation strategy, usually to draw attention to the need for government support for crossing the ‘valley of death’ between proof of concept and viable business model. Figure 14 tentatively proposes a parallel scale of ‘societal readiness levels’ for evaluating the social transition discussed in this issue. The technologies required to deliver material demand reduction already exist, but while scenarios have been developed to show its potential we have very little experience of even early stage demonstrators. To begin the process of moving towards ‘normal practice’ a priority for now must be to develop practical, physical demonstrations of living well with reduced material demand. The opportunity to make such demonstrations is available to everyone, whether at home or at work and the highest priority is therefore to find and support the social entrepreneurs who will create the basis for experience.

## 4. An outline research agenda

The process leading to this issue has raised many opportunities for future research. Some of the most compelling options for further work include:

— Identifying the means to develop and communicate evidence about the environmental impacts of material production and the opportunity to live well with less in what was called in 2016 the ‘post-truth’ world of unedited social media. Related to this, what might be the role of ‘civics’ in creating public connection to decision-making about responses to climate change.
— Developing economic insights into questions that cannot be addressed within the neo-classical model but with the same level of rigour, to be useful as a basis for policy evaluation. These include the questions of how mitigation options influence inequality, which economic activities can show increasing returns to capital and labour without needing increased energy input and what instruments other than taxation may be deployed to support mitigation.
— Exploring whether contemporary manufacturing can learn more from craft approaches to production about work and goods, to re-enable a responsive human-scale production with tools and jobs that enhance creativity (as opposed to machines that eliminate it) and a nearer connection between producer and user?
— Pursuing questions raised in this issue about the meaning of products and human expression in work. Is it possible to explore features of the quality of human experience to reveal more about what we lose as our lives are commoditized and the benefits to well-being of swinging the choices in figure 3 from more influenced to more independent?
— Following the wrong assumptions of Keynes' advice in 1931, exploring how could other patterns of paid and unpaid work, employment and leisure lead to equally or more satisfying lives than those which today tend towards an ever greater intensity of leisure spending?
— Developing and reporting government and corporate metrics to provide a more objective reflection of their impact on society and on present and future energy demand.
— Questioning whether there are insights from experience of material aspiration, relationship, ownership and usership from across time and among cultures that might support reduced demand in contrast with the commoditization of today's developed economies? For example, are there lessons from grass-roots eco-movements or from the adaptation and innovation that occurs in the face of economic stagnation such as that experienced recently in Greece? What else might define the ‘quality’ of material experience?
— Evaluating the literature on sustainable consumption which is largely focused on households to explore whether it provides insights into materially intensive purchases made in business-to-business transactions such as commissioning new office buildings or industrial equipment?
— Exploring whether a low-demand economy characterized by high employment (to compensate for the substitution away from energy) or by low employment (as individuals choose leisure over labour as discussed in §2b)? Are we tackling a problem of scarcity (because we are limiting the use of energy as a key factor of production) or abundance (because we need non-carbon intensive outlets for our demand and because we are not dissipating our surpluses but reinvesting them causing further growth)?

Material demand reduction is a necessary but challenging component of climate mitigation strategy. The starting point of this overview was that the three dominant stakeholders of figure 2 are ‘locked-in’ to inflexible positions, each unable to initiate change until the others request it. However, the outcome of §2 is that breaking the lock-in requires a re-conceptualization of the problem which releases new alignments and alliances. A collective discussion about job design and quality of life for example might be in the interests of all three stakeholders and would allow discussion of change for reasons outside those that create today's apparently fixed positions. The need for such new alignments and alliances is equally revealed by the recommendations in §§3 and 4 about present actions and future research: new configurations are required for us to initiate change and extend our ability to cast new light on this agenda. As one of many early shoots of hope, the meeting and process behind this special issue were conducted with an impressive openness to the possibility of new learning from a collaborative exploration of a common goal. Redirecting our attention to collaborative problem solution in this way must be central to achieving the widely accepted goal of a more sustainable future.
